# The F220C and F45L rhodopsin mutations identified in retinitis pigmentosa patients do not cause pathology in mice

**DOI:** 10.1038/s41598-020-64437-y

**Published:** 2020-05-05

**Authors:** Tylor R. Lewis, Camilla R. Shores, Martha A. Cady, Ying Hao, Vadim Y. Arshavsky, Marie E. Burns

**Affiliations:** 1 Department of Ophthalmology, Duke University Medical Center, Durham, NC 27710 United States; 2 Department of Cell Biology and Human Anatomy, University of California, Davis, CA 95616 United States; 3 Center for Neuroscience and Department of Ophthalmology & Vision Science, University of California, Davis, CA 95616 United States; 4 Department of Pharmacology and Cancer Biology, Duke University Medical Center, Durham, NC 27710 United States

**Keywords:** Retina, Enzyme mechanisms, Cellular neuroscience

## Abstract

Retinitis pigmentosa is a retinal degenerative disease that leads to blindness through photoreceptor loss. Rhodopsin is the most frequently mutated protein in this disease. While many rhodopsin mutations have well-understood consequences that lead to cell death, the disease association of several rhodopsin mutations identified in retinitis pigmentosa patients, including F220C and F45L, has been disputed. In this study, we generated two knockin mouse lines bearing each of these mutations. We did not observe any photoreceptor degeneration in either heterozygous or homozygous animals of either line. F220C mice exhibited minor disruptions of photoreceptor outer segment dimensions without any mislocalization of outer segment proteins, whereas photoreceptors of F45L mice were normal. Suction electrode recordings from individual photoreceptors of both mutant lines showed normal flash sensitivity and photoresponse kinetics. Taken together, these data suggest that neither the F220C nor F45L mutation has pathological consequences in mice and, therefore, may not be causative of retinitis pigmentosa in humans.

## Introduction

Retinitis pigmentosa (RP) is a retinal degenerative disease characterized by a progressive loss of photoreceptors^1^. With a worldwide prevalence of about 1 in 4,000 people, there are an estimated 2 million people affected^2^. Approximately one third of autosomal dominant RP (adRP) cases are caused by mutations in the G protein-coupled receptor (GPCR) rhodopsin, with over 150 different causative mutations currently identified^3^. The majority of these mutations lead to defects in rhodopsin folding, trafficking, chromophore binding or transducin activation; yet, there are mutations that are currently unclassified with no known biochemical or cellular defects^4^.

One unclassified rhodopsin mutation, F220C, was described in 1993^5^ in a study analyzing the sequences of rhodopsin genes from 88 patients/families with a positive family history of adRP. Another retinitis pigmentosa patient bearing the F220C mutant allele was subsequently identified^6^. However, a different study^7^ found that a similar F220L rhodopsin mutation did not co-segregate with adRP. Further, both F220C and F220L mutant rhodopsins have been predicted to have normal protein stability and folding in a computational study^8^, although more recent work found that the F220C mutant has a minor trafficking defect in mammalian cell culture^9^.

Another unclassified rhodopsin mutation, F45L, was first described in 1991^10^ in a study analyzing 161 unrelated patients with adRP, where it was present in a single patient and not in any of 118 normal subjects. Seven family members of this patient were subsequently analyzed and the F45L mutation completely co-segregated with disease in all of them (including four affected and three unaffected family members). The presence of the F45L mutation was subsequently identified in a 34 year-old adRP patient (although the defects in visual function reported in this patient were relatively mild)^11^, a 73 year-old patient with severely affected vision^12^ and two additional patients whose age and pathology were not reported^6^. However, there are two observations that conflict with reports that the F45L allele is pathogenic. First, an adRP patient bearing the F45L mutation inherited this mutation from an asymptomatic father^13^. Second, eight carriers of the F45L mutation were all free of any signs of adRP^14^. When analyzed in cell culture, F45L mutant rhodopsin appeared to traffic normally to the plasma membrane^13,15^. One property that F45L rhodopsin shares with F220C rhodopsin is that they both behaved as monomers in *in vitro* assays in which WT rhodopsin behaved as a dimer^16^.

Given the conflicting evidence surrounding the molecular defects and pathogenicity of F220C and F45L rhodopsin mutations, we generated knockin mice bearing each mutation. We conducted a comprehensive analysis of both heterozygous and homozygous mice from each line, which included various microscopic techniques and single cell suction electrode recordings. Our experiments revealed no evidence of photoreceptor degeneration associated with either mutation, and rod photoreceptors of both lines had normal light sensitivities and photoresponse kinetics. These data challenge the causative role of each mutation in human adRP patients.

## Results

### Generation of F220C and F45L knockin mice

**Figure 1:**
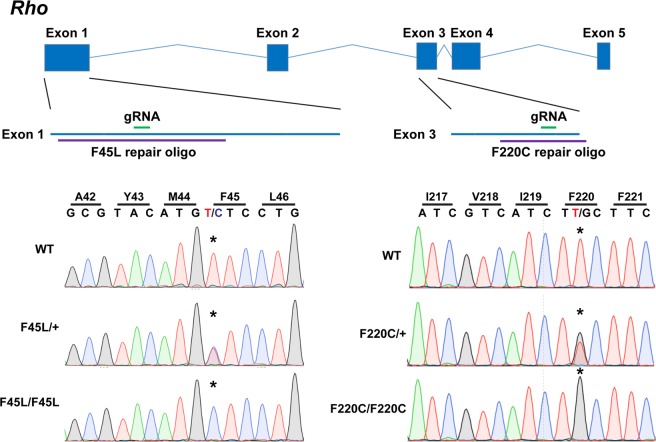
Generation of knockin mouse lines bearing the F220C and F45L rhodopsin mutations. The coding sequence of mouse *Rho* consists of five exons. *(Left)* The F45L mutation is caused by a c.133 T > C mutation in exon 1. A 200-nt long repair oligonucleotide (oligo) was used (purple) in conjunction with the depicted (in green) single small guide RNA (gRNA) and Cas9 mRNA and protein to generate this allele. *(Right)* The F220C mutation is caused by a c.659 T > G mutation in exon 3. A 102-nt long repair oligonucleotide (oligo) was used (purple) in conjunction with the depicted (in green) single small guide RNA (gRNA) and Cas9 protein to generate this allele. Sequencing of DNA from WT, heterozygous, and homozygous mice reveals the correct knockin mutation generated for both the F45L and F220C alleles.

To study the pathogenicity of these rhodopsin mutations, we generated knockin mice using CRISPR/Cas9 methodology for both F220C (c.659 T > G mutation in exon 3 of *Rho*) and F45L (c.133 T > C mutation in exon 1 of *Rho*) rhodopsin mutations (Fig. 1). We used pronuclear injections of short guide RNAs along with both Cas9 mRNA and/or Cas9 protein and repair oligonucleotides containing either the c.659 T > G or c.133 T > C mutation. Potential founders were sequenced to verify the desired nucleotide substitution and the integrity of the remaining gene. Mice were then outcrossed with C57BL/6-J mice for at least three generations before analysis.

### Characterization of the F220C mouse

**Figure 2:**
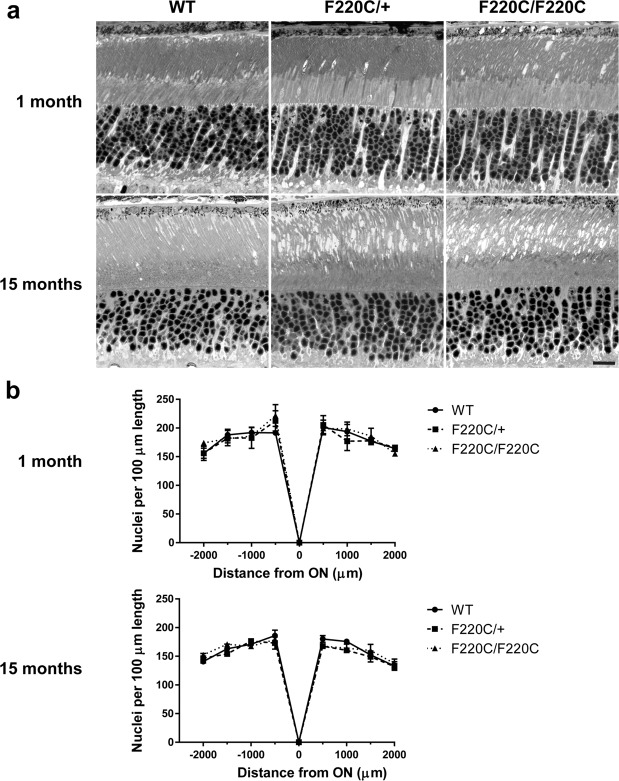
F220C mice do not exhibit photoreceptor degeneration. **(a)** Light microscopy images of 0.5 μm thin retinal plastic sections stained with methylene blue. Depicted are WT, heterozygous (F220C/+), and homozygous (F220C/F220C) retinas at 1 month and 15 months of age. Scale bar is 10 μm. **(b)** The number of photoreceptor nuclei are quantified over a 100 μm length of retina at 500 μm intervals away from the optic nerve (ON) at 1 month and 15 months of age (n = 3 for each genotype at each age). There are no statistically significant differences among genotypes with two-way ANOVA at either age.

To address whether F220C mutant rhodopsin can cause photoreceptor degeneration, we analyzed thin retinal cross-sections from heterozygous F220C knockin mice (F220C/+ ; as in adRP patients), homozygous mice (F220C/F220C) and their WT littermates (Fig. 2). We observed no major difference in either gross photoreceptor morphology or the number of photoreceptor nuclei across these genotypes at either 1 month or 15 months of age. The only subtle difference was the occasional appearance of empty spaces between outer segments of mutant mice. These data show that the F220C rhodopsin mutation does not cause photoreceptor degeneration in mice.

**Figure 3:**
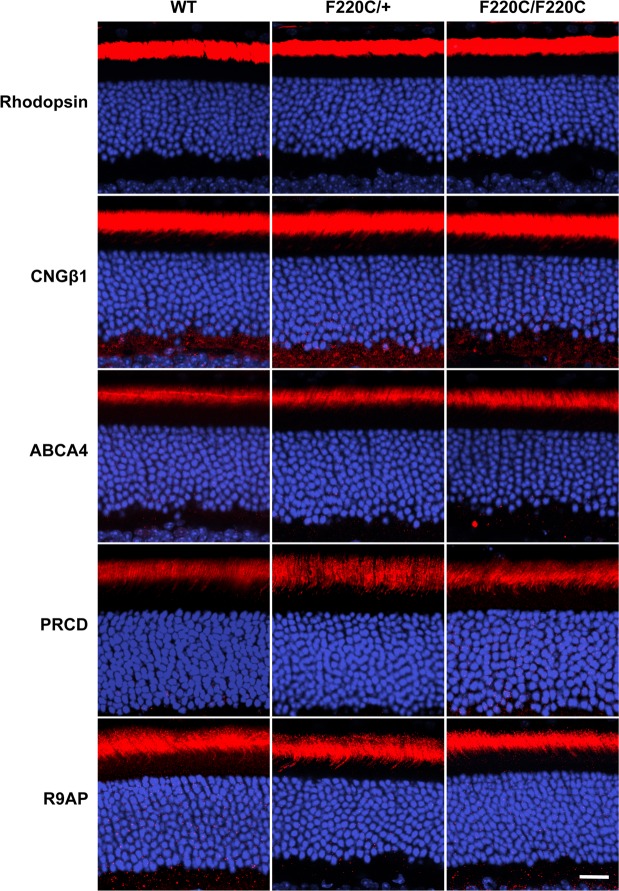
F220C mice do not exhibit mislocalization of photoreceptor outer segment proteins. Immunofluorescent staining of photoreceptor outer segment-specific proteins in WT, heterozygous (F220C/+), and homozygous (F220C/F220C) retinas at 1 month of age. Both disc-specific (rhodopsin, ABCA4, PRCD, R9AP) and outer segment plasma membrane-specific (CNGβ1) proteins are analyzed (red). Nuclei are stained with Hoechst (blue). Scale bar is 10 μm.

We next sought to identify whether there may be any defects in localization of rhodopsin or other outer segment proteins within mutant rods. Figure 3 shows that rhodopsin localization was normal in both hetero- and homozygous mice, as was localization of several other representative outer segment proteins – CNGβ1, ABCA4, R9AP and PRCD – the latter previously shown to rely on rhodopsin for transport to the outer segment^17^.

**Figure 4:**
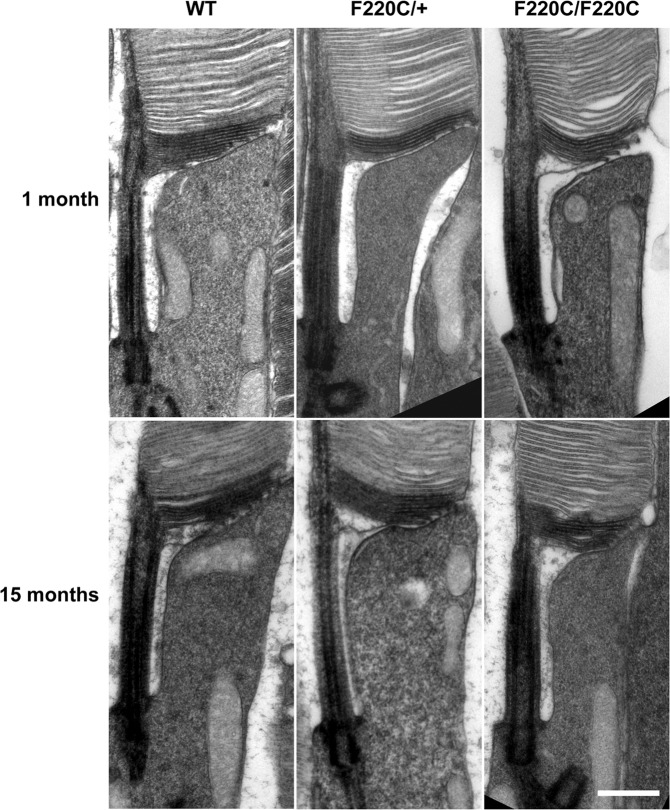
Rod outer segments of F220C mice do not exhibit any gross ultrastructural defects. TEM images of the base of photoreceptor outer segments from WT, heterozygous (F220C/+), and homozygous (F220C/F220C) mice are shown at 1 month and 15 months of age. Sections are stained with tannic acid to intensely label nascent, open discs at the base of the outer segment. Scale bar is 0.5 μm.

To analyze photoreceptor ultrastructure, we performed transmission electron microscopy (TEM) (Fig. 4), focusing primarily on photoreceptor outer segments because rhodopsin is the main protein component of these organelles^18^ and outer segment disc organization is often affected in other mouse adRP models^19,20^. We used tannic acid as a membrane-contrasting reagent, as it preferentially stains membranes of newly formed “open” discs^21^, thereby allowing us to reveal any defect in disc morphogenesis that could be affected by abnormal rhodopsin transport or incorporation into the outer segment membranes. However, TEM analysis showed that outer segments, including new disc membranes, appeared essentially normal in both hetero- and homozygous mice. The only trend we observed was a slightly smaller outer segment diameter of homozygous F220C rods.

**Figure 5:**
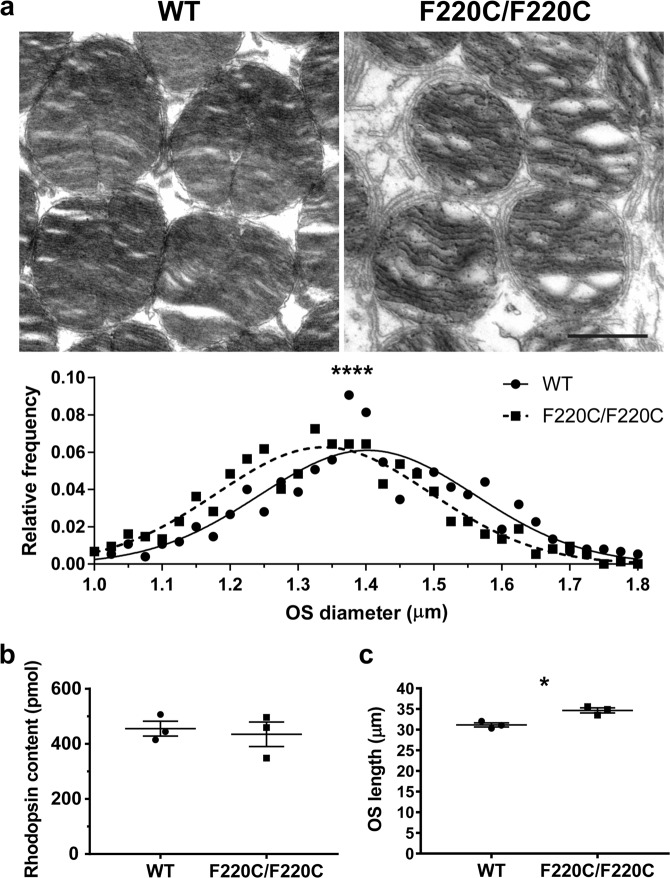
Rod outer segments of F220C mice are slightly thinner and longer. **(a)** TEM of a tangential section through the photoreceptor outer segment layer of WT and homozygous F220C mice. Scale bar is 1 μm. Diameters of 150 outer segments were measured in 5 different mice for a total of 750 outer segments per genotype. Diameters were grouped within 0.02 μm bins to plot the relative frequency of outer segment diameters ranging from 1.0 μm to 1.8 μm. The relative frequency plot is overlaid with Gaussian distribution curves for WT (solid line) and F220C/F220C (dashed line) datasets. Unpaired t-test reveals a statistically significant difference (p < 0.0001) between the outer segment diameters of WT (1.40 ± 0.01 µm) and F220C/F220C (1.34 ± 0.01 µm) mice. **(b)** Total rhodopsin content of dissected eyecups was determined by difference spectroscopy^22^. Unpaired t-test depicts no statistically significant difference (p = 0.7109) in rhodopsin content between WT and homozygous F220C mice at 10 months of age (n = 3 for each genotype). **(c)** Photoreceptor outer segment lengths were measured from five different regions of each of three different mice per genotype at 1 month of age. Unpaired t-test reveals a statistically significant difference (p = 0.011) between the outer segment lengths of WT (34.7 ± 0.6 µm) and F220C/F220C (31.1 ± 0.5 µm) mice.

To follow-up on the latter observation, we performed TEM on tangential sections of outer segments (Fig. 5a). This allowed us to measure the outer segment diameters of a very large number of rods (750 outer segments across 5 WT and homozygous F220C animals) to reliably identify a possible reduction in outer segment size. This quantification revealed that, while the diameter of WT outer segments was 1.40 ± 0.01 µm, the diameter of F220C/F220C outer segments was 1.34 ± 0.01 µm (p < 0.0001). Therefore, the F220C mutation causes a small reduction in outer segment diameter.

To determine how this small reduction in outer segment diameter affected the total rhodopsin content of the retina, we dissected dark-adapted eyecups under dim red light and used difference spectroscopy^22^ to quantify rhodopsin in F220C/F220C and WT eyecups (Fig. 5b). Interestingly, the total rhodopsin content of the F220C/F220C eyecups was no different than that of wild-type (p = 0.7109). We then sought to measure outer segment lengths to determine whether there may be a compensatory effect that offsets the smaller diameter of F220C homozygous outer segments and thus keeps rhodopsin content the same (Fig. 5c). Indeed, when measured on plastic retinal cross-sections, outer segments of F220C/F220C mice did appear slightly longer than in WT mice (34.7 ± 0.6 µm vs. 31.1 ± 0.5 µm; p = 0.011).

**Figure 6:**
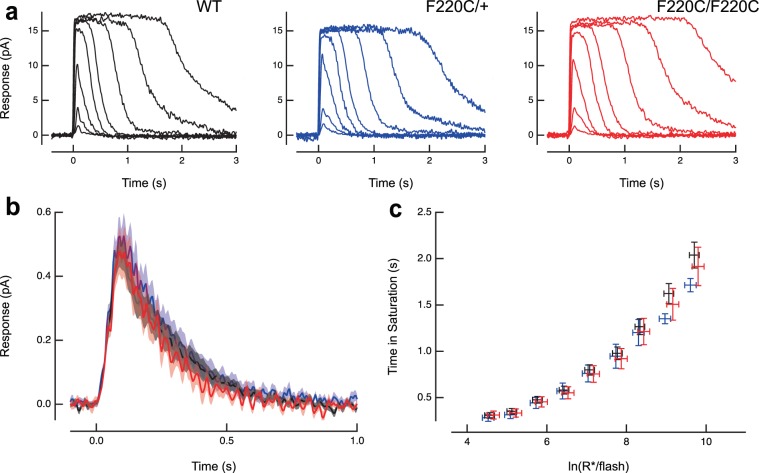
F220C rhodopsin mutant photoreceptors do not exhibit any electrophysiological defects. **(a)** Suction electrode recordings of families of responses to flashes that ranged from 13–51,000 photons/μm^2^ by factors of 2–4. Flashes were delivered at t = 0 s. **(b)** Population average single photon responses calculated from WT (n = 22), F220C/+ (n = 14) and F220C/F220C (n = 11) rods. Light shading represents SEM. **(c)** Relationship between the time that a bright flash response remained in saturation and the natural log of the number of photoexcited rhodopsins (R*) produced by the flash. The initial slope reflects the dominant time constant of recovery for bright flashes, which was the same for mutant and WT rods. Error bars represent SEM.

We next performed single cell suction electrode recordings on rods of 8–13 week old age-matched wildtype, heterozygote and homozygote littermates. Rod responses to calibrated light flashes were very similar in mice of all three genotypes (Table 1; Fig. 6). Rod sensitivity to light was unaffected by the F220C mutation: the amplitude of the response to a single activated rhodopsin (SPR) was indistinguishable between lines and the flash strength needed to generate a half-maximal response (I_o_) was virtually identical (Table 1). The time constants of recovery for dim flash responses (𝜏_rec_) and bright flash responses (𝜏_D_) were likewise indistinguishable (Table 1; Fig. 6). Importantly, there was no significant difference in the effective collecting areas of wild-type and homozygous F220C rods (Table 1), which is likely due to the compensatory effect created by an increase in length and decrease in diameter, as reported above. Taken together, we observed no significant differences between WT, heterozygous and homozygous F220C rods.

**Table 1:** Single cell recordings reveal normal response kinetics in F220C rhodopsin mutant rods.

	Dark current (pA)	SPR amplitude (pA)	Dim flash recovery τ_rec_ (ms)	Dim flash integration time (ms)	Flash Sensitivity I_o_ (photons μm^−2^)	Saturating flash recovery τ_D_ (ms)	Collecting area (µm^2^)
WT	13.3 ± 0.7 (25)	0.50 ± 0.05 (22)	190 ± 12 (23)	225 ± 20 (25)	59 ± 6 (25)	178 ± 10 (25)	0.37 ± 0.08 (22)
F220C/ +	14.3 ± 0.7 (14)	0.59 ± 0.07 (14)	163 ± 22 (14)	227 ± 21 (14)	61 ± 8 (14)	173 ± 17 (14)	0.35 ± 0.04 (14)
F220C/F220C	13.0 ± 0.7 (17)	0.54 ± 0.09 (11)	180 ± 21 (16)	229 ± 22 (16)	58 ± 8 (16)	191 ± 9 (16)	0.38 ± 0.06 (11)

### Characterization of the F45L mouse

**Figure 7:**
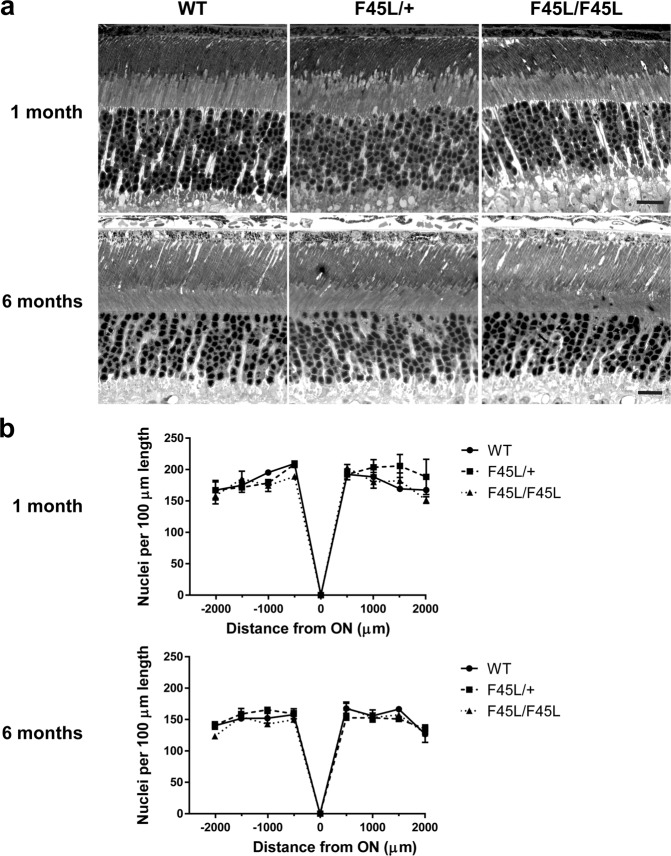
F45L mice do not exhibit photoreceptor degeneration. **(a)** Light microscopy images of 0.5 μm thin retinal plastic sections stained with methylene blue. Depicted are WT, heterozygous (F45L/+), and homozygous (F45L/F45L) retinas at 1 month and 6 months of age. Scale bars are 10 μm. **(b)** The number of photoreceptor nuclei are quantified over a 100 μm length of retina at 500 μm intervals away from the optic nerve (ON) at 1 month and 6 months of age (n = 3 for each genotype at each age). There are no statistically significant differences among genotypes with two-way ANOVA at either age.

**Figure 8:**
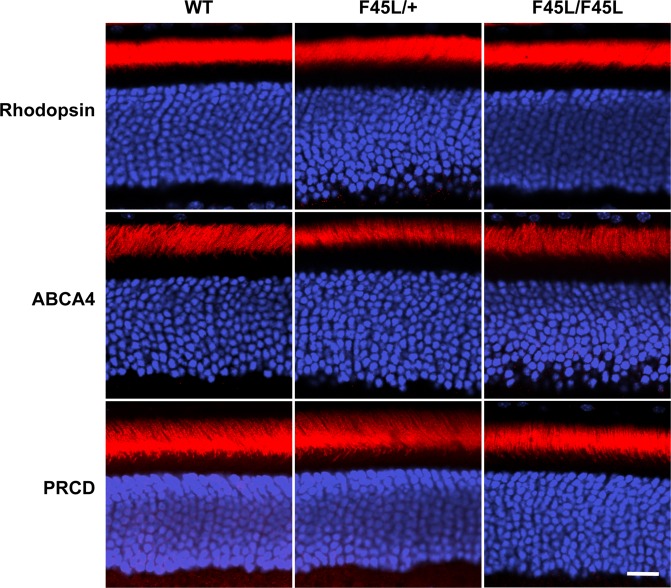
F45L mice do not exhibit mislocalization of photoreceptor outer segment proteins. Immunofluorescent staining of photoreceptor outer segment disc-specific proteins (red) in WT, heterozygous (F45L/+), and homozygous (F45L/F45L) retinas at 1 month of age. Nuclei are stained with Hoechst (blue). Scale bar is 10 μm.

To address whether F45L mutant rhodopsin can cause photoreceptor degeneration, we analyzed thin retinal cross-sections from heterozygous F45L knockin mice (F45L/+ ; as in adRP patients), homozygous mice (F45L/F45L) and their WT littermates (Fig. 7). We observed no major difference in the number of photoreceptor nuclei across these genotypes at either 1 month or 6 months of age. Further, we did not observe any gross morphological differences between genotypes of any age. These data show that the F45L rhodopsin mutation does not cause photoreceptor degeneration in mice. We next investigated localization of rhodopsin and two other representative outer segment proteins (ABCA4 and PRCD) within mutant rods (Fig. 8) and found that their localization was normal in both hetero- and homozygous F45L mice.

**Figure 9:**
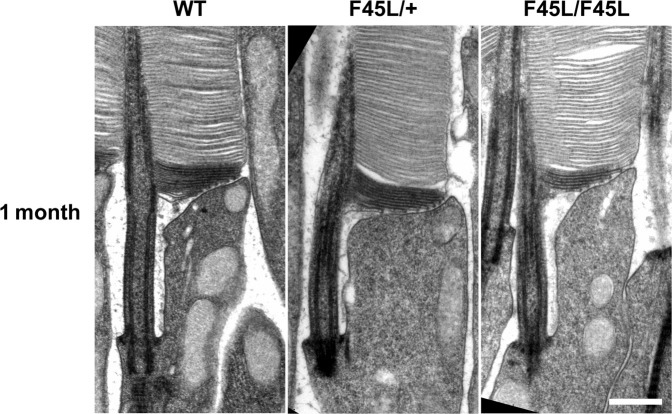
Rod outer segments of F45L mice do not exhibit any gross ultrastructural defects. TEM images of the base of photoreceptor outer segments from WT, heterozygous (F45L/+), and homozygous (F45L/F45L) mice are shown at 1 month of age. Sections are stained with tannic acid to intensely label nascent, open discs at the base of the outer segment. Scale bar is 0.5 μm.

To analyze photoreceptor ultrastructure, we performed transmission electron microscopy (TEM) (Fig. 9), focusing primarily on photoreceptor outer segments. This analysis showed that outer segments, including new disc membranes, appeared normal in both F45L/+ and F45L/F45L mice.

**Figure 10:**
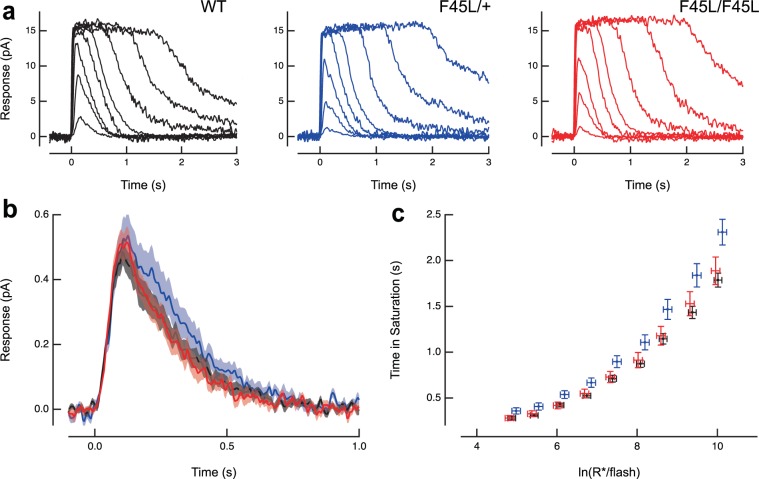
F45L rhodopsin mutant photoreceptors do not exhibit any electrophysiological defects. **(a)** Suction electrode recordings of families of responses to flashes that ranged from 6–50,000 photons/μm^2^ by factors of 2–4. Flashes were delivered at t = 0 s. **(b)** Population average single photon responses calculated from WT (n = 22), F45L/+ (n = 14) and F45L/F45L (n = 19) rods. Light shading represents SEM. **(c)** Relationship between the time that a bright flash response remained in saturation and the natural log of the number of photoexcited rhodopsins (R*) produced by the flash. The initial slope reflects the dominant time constant of recovery for bright flashes, which was the same for mutant and WT rods. Error bars represent SEM.

Lastly, we performed single cell suction electrode recordings on rods of 9–12 week old age-matched wildtype, heterozygote and homozygote littermates, as described above. Rod responses to calibrated light flashes were identical in mice of all three genotypes (Table 2; Fig. 10).

**Table 2:** Single cell recordings reveal normal response kinetics in F45L rhodopsin mutant rods.

	Dark current (pA)	SPR amplitude (pA)	Dim flash recovery τ_rec_ (ms)	Dim flash integration time (ms)	Flash Sensitivity I_o_ (photons μm^−2^)	Saturating flash recovery τ_D_ (ms)	Collecting area (µm^2^)
WT	13.2 ± 0.7 (22)	0.57 ± 0.05 (22)	184 ± 10 (22)	242 ± 13 (22)	44 ± 3 (22)	154 ± 8 (22)	0.50 ± 0.05 (22)
F45L/+	12.7 ± 0.6 (27)	0.61 ± 0.06 (14)	186 ± 10 (27)	247 ± 17 (27)	40 ± 2 (27)	173 ± 8 (26)	0.55 ± 0.07 (17)
F45L/F45L	13.1 ± 06 (30)	0.61 ± 0.04 (19)	177 ± 12 (30)	255 ± 20 (30)	44 ± 2 (30)	170 ± 8 (28)	0.46 ± 0.05 (19)

## Discussion

The data obtained in this study demonstrate that neither the F220C nor the F45L mutation in rhodopsin has pathological consequences in knockin mouse models. The F220C mutation displayed a minor abnormality in the outer segment size, while F45L photoreceptors were completely normal. Since all well-characterized disease-causing rhodopsin mutations lead to photoreceptor degeneration in mice^23,24^, it is unlikely that the F220C and F45L mutations are directly causal in producing adRP. The apparent discrepancy between our mouse data and the notion that these two mutations are causative of adRP can be resolved in a number of ways. As described in the introduction, neither mutation has complete co-segregation with adRP^6,13,14^. The fact that both mutations have been found in healthy humans has been consistently used as an argument against the notion that these mutations are causative of disease, which to a great degree motivated our study.

There are two potential explanations as to why the F45L or F220C mutant alleles were identified in adRP patients. A trivial explanation is that neither allele is pathogenic and they were coincidently present in the individuals analyzed in the original genetic studies^13,25^. This explanation is consistent with a relatively high frequency of these alleles in the general population^25^ and a modest number of subjects analyzed in the original studies. Another possibility is that F45L and F220C are modifying alleles, which interact with other non-rhodopsin mutations to cause retinal degeneration in adRP patients. Examples of gene interactions affecting photoreceptor degeneration and other ciliopathies have been reported in several studies^26–30^. Indeed, most studies of patients bearing the F45L and F220C alleles only analyzed the sequence of the rhodopsin gene^5,10,11,13,14^. Therefore, in conjunction with our data, it is reasonable to suggest that the F220C and F45L substitutions in rhodopsin are most likely polymorphisms that are either benign or modifying alleles.

Despite a lack of photoreceptor degeneration in either F220C or F45L mice, we did observe a small morphological defect associated with the F220C mutation. Outer segments of these mice have a slightly reduced diameter (1.34 µm vs. 1.40 µm in WT). One potential explanation for this phenomenon is a reduced ability of F220C rhodopsin to correctly fold in the biosynthetic membranes, such that the amount of rhodopsin delivered to the outer segment is slightly reduced. Based on the measured reduction of the outer segment diameter and the corresponding reduction in the disc surface area in F220C mice, we estimate that the amount of rhodopsin transported to the outer segment is approximately 92% of WT. This explanation is consistent with a recent finding that the F220C rhodopsin mutant has a minor trafficking defect in cultured mammalian cells, with only 86% of mutant protein trafficked to the plasma membrane^9^.

The phenotype of F220C mice can be compared to that of rhodopsin hemizygous mice whose outer segment diameter is only 1.1–1.2 µm, corresponding to about 60% of the WT outer segment rhodopsin content^31,32^. Rhodopsin hemizygous mice exhibit slow photoreceptor degeneration with 1–2 rows of photoreceptor nuclei reported to be lost by 3 months of age^33^. Together, these results suggest that while photoreceptors can tolerate a small decrease in the rhodopsin content of discs (e.g. the 8% decrease observed in F220C mutant mice), there is some level of reduced rhodopsin content that ultimately causes pathology.

The reduced diameter of F220C homozygous outer segments is offset by an increase in length by approximately 11%, which results in no detectable change in the total rhodopsin content of the retina as measured by difference spectroscopy or collecting area measurements obtained from suction electrode recordings. These data support the idea that the photoreceptor outer segment has some innate capacity to regulate its propensity to capture photons, perhaps related to other reported mechanisms of outer segment length control^34^ or through regulation of disc shedding^35^. Interestingly, such compensation is not observed in the rhodopsin hemizygous mouse whose outer segments are both thinner and shorter than in WT mice^32,33^.

Lastly, a recent study found that both F220C and F45L mutant rhodopsin behave as monomers in *in vitro* reconstitution assays, whereas WT rhodopsin behaves as a dimer. Given that GPCR dimerization has previously been shown to regulate receptor folding^36–41^ and trafficking^42–45^, it is intriguing to believe that the phenotype of the F220C mutant could be due to issues with rhodopsin dimerization. Indeed, the only study to address the effect of disrupted rhodopsin dimerization in intact photoreceptors reported that inhibitory peptides^46^, shown to mildly disrupt rhodopsin dimerization *in vitro*^47^, caused rhodopsin mislocalization^46^. Yet, another recent study in mammalian cells suggested that F220C mutant rhodopsin does not affect its dimerization^9^. While rhodopsin dimerization within disc membranes has been shown through atomic-force microscopy^48^ and cryo-electron microscopy^49^, there is currently no assay for analyzing rhodopsin dimerization during folding and trafficking, precluding us for identifying whether the F220C mutation in rhodopsin does indeed affect its dimerization *in vivo*, and, subsequently, what the role of rhodopsin dimerization in intact photoreceptors is.

## Materials and Methods

### Animals

All methods with regard to mice handling were carried out in accordance with relevant guidelines and regulations. F220C mutant mice were generated through the Duke Transgenic and Knockout Mouse Facility through pronuclear injections of C57BL/6 J mouse embryos with 2 ng/µl of a short guide RNA (5′ TCGTCATCTTCTTCTGCTAT GGG 3′), 8 ng/µl of a 102-nucleotide repair oligonucleotide containing the c.659 T > G mutation to knock-in the F220C allele (5′ T GTC ATC TAC ATG TTC GTG GTC CAC TTC ACC ATT CCT ATG ATC GTC ATC TGC TTC TGC TAT GGC CAG CTG GTC TTC ACA GTC AAG GAG GTA TGA GCA GGG GG 3′), 2 ng/µl Cas9 mRNA, and 2 ng/µl Cas9 protein. Potential founders were genotyped for successful knock-in through sequencing of a 491-base pair PCR product of the rhodopsin locus using primers (5′ GCAGAGCTGCGTGGTCAAGTGG 3′; 5′ CCTTCTGAGTGGTGGCTGACTCC 3′). F45L mutant mice were generated through the Duke Transgenic and Knockout Mouse Facility through pronuclear injections of C57BL/6 J mouse embryos with 200 ng/µl of a short guide RNA (5′ GAACATGTACGCTGCCAGCA TGG 3′), 0.649 ng/µl of a 200-nucleotide repair oligonucleotide containing the c.133 T > C mutation to knock-in the F220C allele (5′ CCC TTC TCC AAC GTC ACA GGC GTG GTG CGG AGC CCC TTC GAG CAG CCG CAG TAC TAC CTG GCG GAA CCA TGG CAG TTC TCT ATG CTG GCA GCG TAC ATG CTC CTG CTC ATC GTG CTG GGC TTC CCC ATC AAC TTC CTC ACG CTC TAC GTC ACC GTA CAG CAC AAG AAG CTG CGC ACA CCC CTC AAC TAC ATC CTG CTC AA 3′), and 8 µM Cas9 protein. Potential founders were genotyped for successful knock-in through sequencing of a 596-base pair PCR product of the rhodopsin locus using primers (5′ GCCTCCACCCGATGTCACC 3′; 5′ CCCTCGAGATTACAGCCTG 3′). Mice bearing successful knock-ins were outcrossed to C57BL/6 J mice for several generations. All mice were genotyped to ensure that they did not contain either the *rd8*^50^ or *rd1*^51^ mutations commonly found in inbred mouse strains. All mice were housed under a 12/12 hour diurnal light cycle. Littermate WT, heterozygous, and homozygous mutant mice were used for all experiments.

### Immunofluorescence

Anesthetized mice were transcardially perfused with a fixative solution containing 4% paraformaldehyde in 80 mM PIPES (pH 6.8), 5 mM EGTA, and 2 mM MgCl_2_. Eyes were enucleated and post-fixed in the same solution for two hours at room temperature. After fixation, dissected eyecups were embedded in 2.5% low-melt agarose (Precisionary) and cut by a Vibratome (VT1200S; Leica) into 100 µm thick slices as described previously^52^. Agarose sections were blocked in PBS containing 5% donkey serum and 0.5% Triton X-100 for 1 h at room temperature before staining with primary antibody in blocking buffer overnight at 4 °C. After primary antibody staining, sections were washed three times in PBS and incubated with secondary antibody in blocking buffer overnight at 4 °C. Finally, sections were washed three times in PBS and nuclei were stained with 10 µg/ml Hoechst (H3569; Thermo Fisher Scientific) for 30 min at room temperature. Finally, sections were washed three times in PBS, and mounted onto slides with Immu-Mount (Thermo) and coverslipped. Images were taken with a confocal microscope (Eclipse 90i and A1 confocal scanner; Nikon) with a 60× objective (1.4 NA Plan Apochromat VC; Nikon) using Nikon NIS-Elements software. Image analysis and processing was performed with ImageJ.

### Antibodies

For immunofluorescence imaging, commercial primary antibodies included: polyclonal goat anti-human ABCA4, 1:2000 (Everest Biotech) and monoclonal mouse anti-bovine RHO (1D4), 1:2000 (Abcam)^53^. The polyclonal rabbit anti-mouse CNGβ1, 1:500, was a generous gift from Dr. Steven Pittler. The polyclonal rabbit anti-mouse R9AP, 1:2500, was a generous gift from Dr. Stefan Heller^54^. The polyclonal rabbit anti-mouse PRCD, 1:2000, was previously generated by the Arshavsky lab^55^. The commercial secondary antibodies included donkey anti-goat, donkey anti-mouse, and donkey anti-rabbit conjugated to Alexa Fluor 488 or 568, 1:1000 (Invitrogen).

### Histological techniques and transmission electron microscopy

Fixation and processing of mouse eyes for light microscopy of plastic sections was performed as described previously^21^. Anesthetized mice were transcardially perfused with 2% paraformaldehyde, 2% glutaraldehyde, and 0.05% calcium chloride in 50 mM MOPS (pH 7.4) resulting in exsanguination. Enucleated eyes were fixed for an additional 2 h in the same fixation solution at room temperature. To obtain semi-thin plastic retinal sections, eyecups were cut in half through the optic nerve, dehydrated with ethanol, and embedded in EMbed 812 (Electron Microscopy Sciences). Embedded retinal cross sections were cut through the optic nerve in 500 nm slices and stained with methylene blue for light microscopy as previously described^56^. Images were taken with a confocal microscope (Eclipse 90i and A1 confocal scanner; Nikon) with a 60× objective (1.4 NA Plan Apochromat VC; Nikon) using Nikon NIS-Elements software. Image analysis and processing was performed with ImageJ.

For TEM involving tannic acid staining, eyes were fixed as described above. Eyecups were dissected from fixed eyes, embedded in 2.5% low-melt agarose (Precisionary), and cut into 200 µm thick slices on a Vibratome (VT1200S; Leica)^21^. Agarose sections were stained with 1% tannic acid (Electron Microscopy Sciences) and 1% uranyl acetate (Electron Microscopy Sciences), gradually dehydrated with ethanol, and infiltrated and embedded in Spurr’s resin (Electron Microscopy Sciences). For TEM involving tangential sections of outer segments, eyes were fixed as described above. Dissected eyecups were flatmounted, dehydrated with ethanol, and embedded in EMbed 812 (Electron Microscopy Sciences). For all TEM, 70 nm sections were cut, placed on copper grids, and counterstained with 2% uranyl acetate and 3.5% lead citrate (19314; Ted Pella). The samples were imaged on a JEM-1400 electron microscope (JEOL) at 60 kV with a digital camera (Orius; Gatan).

### Suction electrode recordings

Animals used for electrophysiological analysis were cared for and handled in accordance with a protocol approved by the Institutional Animal Care and Use Committees of UC Davis. Suction electrode recordings from the outer segments of intact mouse rods were performed as previously described^57^. Briefly, mice 2–3 months of age were dark-adapted overnight, euthanized, and their retinas were dissected and stored on ice in L-15 medium supplemented with 10 mM glucose. Recordings were performed in oxygenated, bicarbonate buffered Locke’s solution supplemented with 10 mM glucose at 35–37 °C. A suction pipette containing HEPES-buffered Locke’s solution (pH 7.4) recorded the electrical responses to brief (10 ms, 500 nm) flashes of calibrated strength, which were amplified (Axopatch 200B; Molecular Devices), filtered at 30 Hz with an eight-pole Bessel (Frequency Devices), and digitized at 200 Hz using custom-written acquisition procedures in IgorPro (Wavemetrics). Responses to a large number (>30) of dim flashes were averaged and used to determine the mean time to peak and integration time (time integral of the response divided by the peak amplitude). Variance-to-mean analysis of the dim flash responses was used to calculate the average single-photon response (SPR) and effective collecting areas for each cell, and saturating flash responses were used to calculate the dominant time constant of recovery (τ_D_), as previously described^57–59^.

### Rhodopsin difference spectra

Mice were dark-adapted overnight before removal of cornea and lens during dim red light. Eyecups were lysed in 600 µl of 2% octyl glucoside with protease inhibitor (cOmplete; Sigma Aldrich) and sonicated 3 times each of 5 seconds. Lysates were centrifuged at 10,000 × g for 10 minutes and the rhodopsin concentration of the supernatant was measured by difference spectroscopy^22^. Hydroxylamine (25 mM; pH 7.5) was added to 100 μl of lysate and the initial spectra recorded from 300 to 700 nm using a DU800 spectrophotometer (Beckman). The cuvette sample was then bleached with a 100-W halogen lamp for 60 s before taking the second spectrum reading. The difference in the absorbance at 500 nm was used to calculate rhodopsin concentration, using the extinction coefficient of 40,500 M^−1^ ·cm^−1^.

### Quantification and statistical analysis

To assess photoreceptor degeneration, we counted photoreceptor nuclei in 100 μm boxes at 500 μm intervals from the optic nerve spanning 2000 μm in each direction for three mice of each genotype at each age, as previously described^60^. Two-way ANOVA (with respect to genotype and location) was performed to assess any statistical differences between genotypes. To measure photoreceptor diameter, the diameter of 150 outer segments was measured in each of 5 mice for each genotype for a total of 750 outer segments measured per genotype. A normal Gaussian distribution was fit to each genotype. Unpaired, two-tailed t-test (assuming equal variances) t-test was performed to assess any statistical differences between genotypes. To determine the total rhodopsin content of eyecups, difference spectra were taken from each of three dissected eyecups per genotype. Unpaired, two-tailed t-test (assuming equal variances) was performed to assess any statistical differences between genotypes. To quantify outer segment length, the lengths of outer segments in five regions across the retina were measured of each of three different retinal sections per genotype. Unpaired, two-tailed t-test (assuming equal variances) was performed to assess any statistical differences between genotypes. For all experiments, significance is labeled as *p ≤ 0.05, **p ≤ 0.01, ***p ≤ 0.001, ****p ≤ 0.0001. Non-significant values are unlabeled. Data is represented as mean ± standard error of the mean.

## Data Availability

All data generated or analyzed during this study are included in this published article and available from authors on reasonable request.
